# Palmitic acid causes increased dihydroceramide levels when desaturase expression is directly silenced or indirectly lowered by silencing AdipoR2

**DOI:** 10.1186/s12944-021-01600-y

**Published:** 2021-11-28

**Authors:** Mario Ruiz, Marcus Henricsson, Jan Borén, Marc Pilon

**Affiliations:** 1 Department Chemistry and Molecular Biology, Univ. Gothenburg, Box 462, 405 30 Gothenburg, Sweden; 2 Department Molecular and Clinical Medicine/Wallenberg Laboratory, Institute of Medicine, Univ. of Gothenburg, 405 30 Gothenburg, Sweden

**Keywords:** Ceramide, Adiponectin receptor, Phospholipid, Lipidomics, Desaturase, Palmitic acid, Dihydroceramide

## Abstract

**Background:**

AdipoR1 and AdipoR2 (AdipoRs) are plasma membrane proteins often considered to act as adiponectin receptors with a ceramidase activity. Additionally, the AdipoRs and their yeast and *C. elegans* orthologs are emerging as membrane homeostasis regulators that counter membrane rigidification by promoting fatty acid desaturation and incorporation of unsaturated fatty acids into phospholipids, thus restoring fluidity.

**Methods:**

Using cultured cells, the effects of AdipoR silencing or over-expression on the levels and composition of several sphingolipid classes were examined.

**Results:**

AdipoR2 silencing in the presence of exogenous palmitic acid potently causes increased levels of dihydroceramides, a ceramide precursor in the de novo ceramide synthesis pathway. Conversely, AdipoR2 over-expression caused a depletion of dihydroceramides.

**Conclusions:**

The results are consistent with AdipoR2 silencing leading to increased intracellular supply of palmitic acid that in turn leads to increased dihydroceramide synthesis via the rate-limiting serine palmitoyl transferase step. In agreement with this model, inhibiting the desaturase SCD or SREBF1/2 (positive regulators of SCD) also causes a strong increase in dihydroceramide levels.

**Supplementary Information:**

The online version contains supplementary material available at 10.1186/s12944-021-01600-y.

## Introduction

**Fig. 1:**
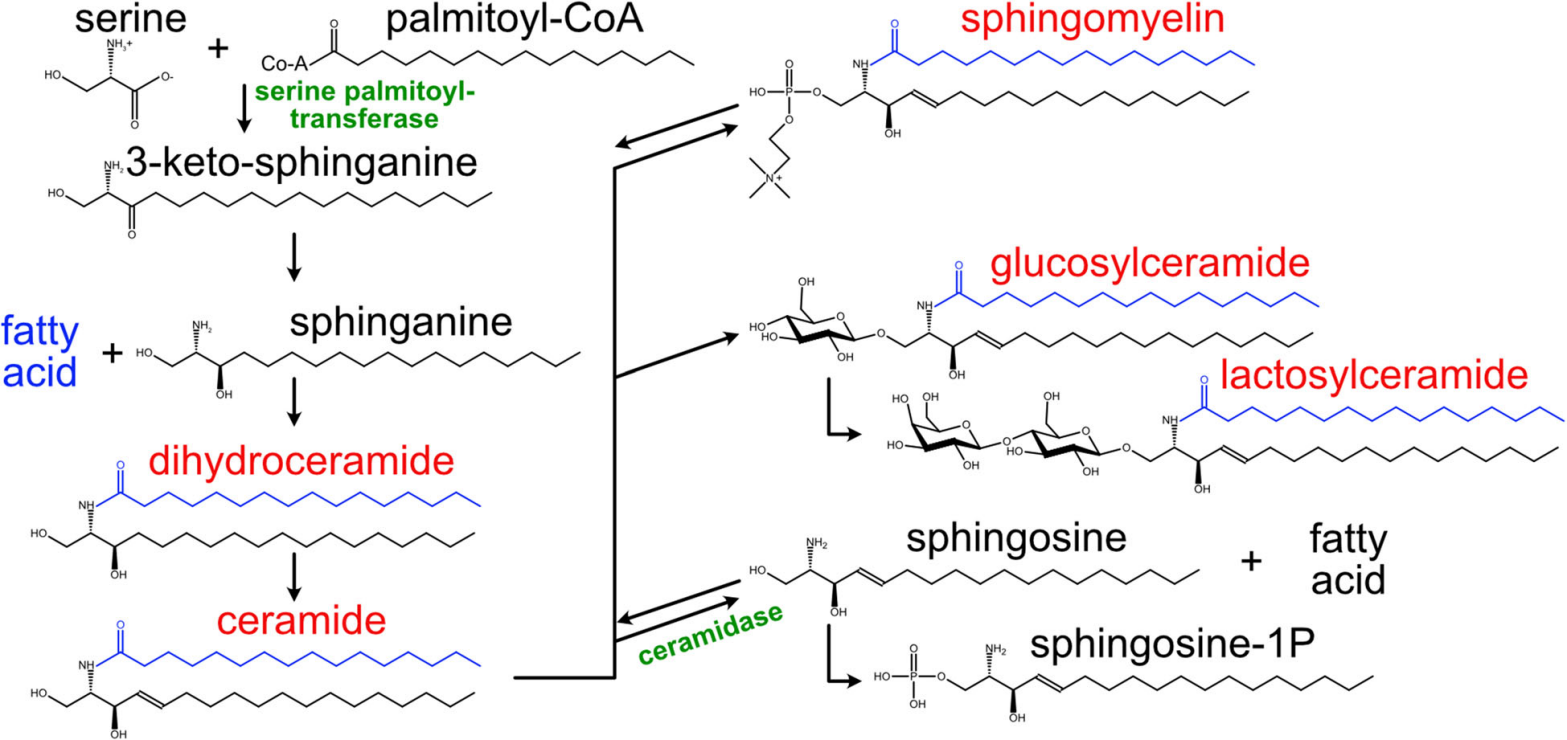
Overview of sphingolipid metabolism. Species in red were measured in the present study both in terms of their total abundance and in terms of their fatty acid composition; a palmitate is shown in blue, but other fatty acids may be present. Two enzymes of particular interest are indicated in green: serine palmitoyltransferase is the rate limiting step in de novo ceramide synthesis while a ceramidase activity has been attributed to the AdipoRs

Ceramides are membrane lipids usually found in very small amounts (< 1 mol%) in cell membranes, although their concentration may increase under stress conditions [1]. A ceramide is composed of a sphingosine base and a conjugated fatty acid that is usually a saturated fatty acid (SFA) but can also be a monounsaturated fatty acid (MUFA; Fig. 1). Ceramides are therefore very hydrophobic and water insoluble, and tend to gather into distinct thick and rigid domains (often referred to as “rafts”) with negative curvature within phospholipid bilayers. Increasing ceramide levels leads to more/larger rafts, which can impact signaling via proteins that sort to the rafts because of their thickness and hydrophobic nature [1]. Ceramides can also signal directly via interacting proteins, such as ceramide-activated protein phosphatases [2]. Additionally, membranes tend to be permeable at the boundaries between the rigid ceramide domains and the rest of the more fluid membrane, which can also contribute to their toxicity [1]. Elevated ceramide levels in various models are associated with impaired insulin signaling [3–5], apoptosis [6] and metabolic syndrome complications [7–9]. Note however that in many studies, the elevated ceramides are also accompanied by elevated levels of SFA, which complicates interpretations especially given that SFA toxicity can occur independently of ceramides [10, 11]. The concentration of ceramides is tightly regulated, usually by converting them into less toxic forms with larger hydrophilic head groups (e.g. sphingomyelin, glucosylceramide or lactosylceramide; Fig. 1).

AdipoR1 and AdipoR2 are members of the PAQR (Progestin and AdipoQ Receptors) protein family that are emerging as regulators of fatty acid desaturation and membrane homeostasis [12]. These proteins have seven transmembrane domains with a cytosolic N-terminus and their C-terminus pointing towards the extracellular space [13]. The AdipoRs were initially identified as adiponectin receptors [14], but recent experimental evidence based on studying the yeast [15–19] and *C. elegans* [20–28] homologs, and the AdipoRs themselves [23–27, 29, 30], suggests that their primary cellular function is in membrane homeostasis and is independent of adiponectin (reviewed in [12]). In particular, the AdipoRs react to membrane rigidification, e.g. when the SFA levels increase in phospholipids, by stimulating expression of desaturases, namely SCD, FADS1 and FADS2 until fluidity is restored [12, 23–26, 28–30].

PAQR proteins are part of the larger CREST family of hydrolases [31], and the X-ray analysis of AdipoR1 and AdipoR2 crystals show that they each forms a barrel structure within the membrane that is closed on its extracellular-facing side but open towards the cytoplasm where it could accommodate entry of a fatty acid or fatty acid-like substrate [32, 33]. Pioneering studies of the yeast IZH2 (an AdipoR homolog) indicated that it is a ceramidase and that its function can be partially replaced by the human AdipoRs [34–36]. In vitro assays also showed that recombinant human AdipoR proteins have a ceramidase activity, though with an extremely slow reaction rate, which raises the possibility that they may have other preferred substrates [33]. Importantly, overexpression of AdipoR1 or AdipoR2 in mice causes increased ceramidase activity accompanied by improved metabolic health [37] while mouse embryonic fibroblasts lacking both AdipoRs show reduced ceramidase activity [38].

Increasing the intracellular supply of palmitic acid (PA) potently increases ceramide levels, likely because PA is limiting for ceramide synthesis where the rate-limiting step is carried out by the enzyme serine palmitoyltransferase, which combines PA and serine into 3-keto-sphinganine (see pathway in Fig. 1) [39–42]. Ceramide levels can also be affected by modulating the conversion rates between ceramides and their derivatives, such as sphingomyelins, glucosylceramides or lactosylceramides (Fig. 1) There are therefore at least three different hypotheses that could explain the inverse correlation between AdipoR expression levels and ceramide levels: 1) The putative AdipoR ceramidase activity is potent enough to deplete bulk levels of ceramides within cells and organs; 2) AdipoR signaling regulates the conversion of sphingomyelin or other complex ceramides into ceramides; and 3) AdipoR signaling promotes PA desaturation, resulting in reduced de novo ceramide synthesis. In the present study, lipidomics data were analyzed with a focus on ceramide quantification from several experiments in which the expression of the AdipoRs and other lipid metabolism genes were experimentally manipulated. The results suggest that the increased in dihydroceramide levels observed when AdipoR2 is silenced is mostly due to the fact that SCD is also silenced under these conditions, which impacts ceramide de novo synthesis.

## Methods

### Cell culture

HEK293 and HepG2 cells were obtained from the American Type Culture Collection (ATCC, Manassas, USA) and grown in DMEM containing glucose 1 g/l, pyruvate and GlutaMAX and supplemented with 10% fetal bovine serum, 1% non-essential amino acids, HEPES 10 mM and 1% penicillin and streptomycin (all from Life Technologies, Carlsbad, USA) at 37 °C in a water-humidified 5% CO_2_ incubator. Cells were subcultured twice a week at 90% confluence. TrypLE Express reagent (Gibco, Carlsbad, USA) was used to detach HEK293 and Accutase (GE Healthcare, Chicago, USA) was used to detach HepG2 cells. All cell types were cultivated on treated plastic flasks and multidish plates (Nunc, Roskilde, Denmark) and for HepG2 coated with 0.1% porcine gelatin (Sigma-Aldrich, St. Louis, USA).

### Fatty acid (FA) treatment

PA was dissolved in dimethyl sulfoxide and d31-PA in ethanol (Sigma-Aldrich, St. Louis, USA). The fatty acids were mixed in 0.5% fatty acid-free bovine serum albumin (BSA; Sigma-Aldrich, St. Louis, USA) in serum-free medium for 15 min at room temperature. The resulting molecular ratios of BSA to PA were 1:5.3 in experiments that used 400 μM PA, 1:2.65 in experiments that used 200 μM PA and 1:1.325 when using 100 μM PA. Cells were then incubated in serum-free media containing BSA alone (basal media) or BSA/FA conjugates for 24 h before analysis, except for the AdipoR2 over-expression experiment where PA treatment was 6 h as previously published [27].

### siRNA treatment

siRNAs were obtained from Dharmacon (Lafayette, USA): Non-targeting (NT) D-001810–10, AdipoR1 J-007800-10, AdipoR2 J-007801–10, SCD J-005061-07, FADS2 J-008211-09, ACSL4 J-009364-05, PEMT J-010392-05, SREBF1 J-006891-05 and SREBF2 J-009549-05. Transfection of 25 nM siRNA in complete medium using Viromer Blue was carried out according to the manufacturer’s instructions 1× (Lipocalyx, Halle, Germany). Knockdown expression of the target genes was verified by quantitative PCR (qPCR) to be < 25% of control 48 h after transfection as previously shown for the samples used in the present manuscript [29, 30].

### AdipoR2 over-expressing line generation

The AdipoR2-expressing pIREShyg2-HA-hAdipoR2-cMYC [27] was transfected in HEK293 cells using Viromer Red (Lipocalyx, Halle, Germany), adjusting the concentration to 1/10 of Viromer Red reagent and 1/5 plasmid as per the 1X original protocol. Cells stably overexpressing AdipoR2 were selected using Hygromycin B (Tocris, Bristol, USA) at 200 μg/ml and over-expression was verified by Western-blot and qPCR.

### Quantitative PCR (qPCR)

Total cellular RNA was purified with an RNeasy Kit (Qiagen, Hilden, Germany) and quantified using a ND-100 NanoDrop spectrophotometer (Thermo Fisher Scientific). cDNA was generated using random hexamers and a High-Capacity cDNA Reverse Transcription Kit (Applied Biosystems, Waltham, USA) or RevertAid H Minus First Strand cDNA (ThermoScientific, Carlsbad, USA). qPCR using HOT FIREpol EvaGreen qPCR Super-mix (Solis Biodyne, Tartu, Estonia) and standard primers was performed in a CFX Connect thermal cycler (Bio-Rad, Hercules, USA). Samples were quantified in triplicates and relative gene expression was calculated using the ΔΔCT method [43]. Quantification of the reference gene PPIA was used to normalize for variations in RNA input. Primers used are described in [29, 30]. Primers used in this study were: AdipoR1-For (CCATCTGCTTGGTTTCGTGC) and -Rev (AGACGGTGTGAAAGAGCCAG), AdipoR2-For (TCATCTGTGTGCTGGGCATT) and -Rev (CTATCTGCCCTATGGTGGCG), PPIA-For (GTCTCCTTTGAGCTGTTTGCAG) and -Rev (GGACAAGATGCCAGGACCC), SCD-For (TTCGTTGCCACTTTCTTGCG) and -Rev (TGGTGGTAGTTGTGGAAGCC), FADS-1-For (TGGCTAGTGATCGACCGTAA) and –Rev (GGCCCTTGTTGATGTGGAAG), FADS-2-For (GGGCCGTCAGCTACTACATC) and –Rev (ACAAACCAGTGGCTCTCCAG), PEMT-For (AGCTTCTTTGCACTGGGGTT), PEMT-Rev (GGGCTGGCGTGCATGAT), SREBF1-For (GACCTCGCAGATCCAGCAG), SREBF1-Rev (ATAGGCAGCTTCTCCGCATC), SREBF2-For (GTGCTGTTCCTGACTCCCTG) and SREBF2-Rev (CAGCCTTCTTCTTGGCCTGA).

### Protein samples and Western blotting

Cellular proteins were extracted, separated by gel electrophoresis, transferred to nitrocellulose membranes and blocked with 5% nonfat dry milk as previously described [27]. The blots were then incubated overnight at 4 °C with primary antibodies, as follows: rabbit monoclonal anti-HA antibody (C29F4; Cell signaling) 1:5000 dilution, rabbit anti-GAPDH antibody (14C10; Cell Signaling, Danvers, USA) or rabbit polyclonal anti-AdipoR2 (1:1000, described in [44]). Blots were then washed with PBS-T and incubated with a swine anti-rabbit IgG/HRP (1:3000 dilution; Dako, Glostrup, Denmark) or goat anti-mouse HRP (1:3000, Dako, Glostrup, Denmark) then washed again with PBS-T. Blots were developed using an ECL kit (Immobilon Western; Millipore), and the signals documented using a digital camera (VersaDoc; Bio-Rad, Hercules, USA). The membranes were then stripped and reprobed with anti-GAPDH (14C10) rabbit IgG (1:2500 dilution; Cell Signaling, Danvers, USA), which served as a loading control. The PageRuler Plus prestained protein ladder was used to assess molecular weight (Thermo Fisher Scientific, Carlsbad, USA).

### Lipidomics

At least three independent replicates for each condition were prepared from cells cultivated in basal media or in the presence of 200 or 400 μM PA for 24 h or 100 μM d31-PA prior to harvesting. To extract lipids, cells were pelleted then sonicated for 10 min in methanol then extracted according to published methods [45]. Internal standards were added during the extraction. Phospholipids and sphingomyelins were measured using direct infusion mass spectrometry. For phospholipids, a part of the lipid extracts were evaporated and reconstituted in chloroform:methanol [1:2] with 5 mM ammonium acetate. This solution was infused directly (shotgun approach) into a QTRAP 5500 mass spectrometer (Sciex, Framingham, USA) equipped with a with a TriVersa NanoMate (Advion Bioscience, Ithaca, USA) as described previously [46]. For the analysis of sphingolipds, lipid extracts were first evaporated and exposed to alkaline hydrolysis (0.1 M KOH in methanol for 60 min) in order to remove phospholipids. For phosphatidylcholines (PC) and sphingomyelins mass spectra were obtained in precursor ion scanning mode using the phosphocholine headgroup (*m/z* 184.1) as fragment ion. For phosphatidylethanolamines neutral loss scanning of *m/z* 141.1 was used [47, 48]. Sphingolipids (except sphingomyelins) were measured using ultra performance liquid chromatography coupled to tandem mass spectrometry according to previous publication [49]. The data were analyzed using the LipidView and MultiQuant softwares (Sciex, Framingham, USA). Quantification of each sphingolipid species is expressed as a ratio of their abundance over that of the total PC amount, i.e. as pmol sphingolipid/nmol PC, and the complete lipidomics data is available in Supplemental Table S1. The software Qlucore Omics Explorer (Qlucore, Lund, Sweden) was employed for multivariate analysis.

### Statistical analysis

Student’s t-tests were used to determine significance in cases where samples from a single condition were compared with control samples. One-way ANOVA was used when multiple conditions were compared within one experiment, and a post hoc Dunnett’s test was used to identify conditions that differed significantly from the control. Cochran’s Q test of proportion was used to establish differences in the ceramide and dihydroceramide increases across multiple independent experiments. *P* values are noted as follows: * *P* < 0.05, ** *P* < 0.01 and ***P < 0.001. For heat maps, only lipid species that showed significant differences among the samples based on ANOVA and with corrected *P* values of q < 0.05 are shown.

## Results

### Overview of the experiments and data analysis

For the present study, unpublished lipidomics data were analyzed from several experiments carried out during the period 2017 to 2020, with a focus on ceramide quantification. The sphingolipids quantified in the present study were ceramides, dihydroceramides, glucosylceramides, lactosylceramides and sphingomyelins, though not all sphingolipids were quantified in all experiments. There are several ways to present lipidomics data. For this study, all sphingolipids were quantified as a ratio of their abundance over that of the total phosphatidylcholine amount, i.e. as pmol sphingolipid/nmol PC. For heat maps and principal component analyses (PCA), the data from each experiment was further normalized to the average value of its control NT (non-targeting) siRNA + PA treatment (the only condition used in all experiments); this allows comparisons across experiments and makes it easy to identify lipid species showing pronounced fold-changes across treatments.

**Fig. 2:**
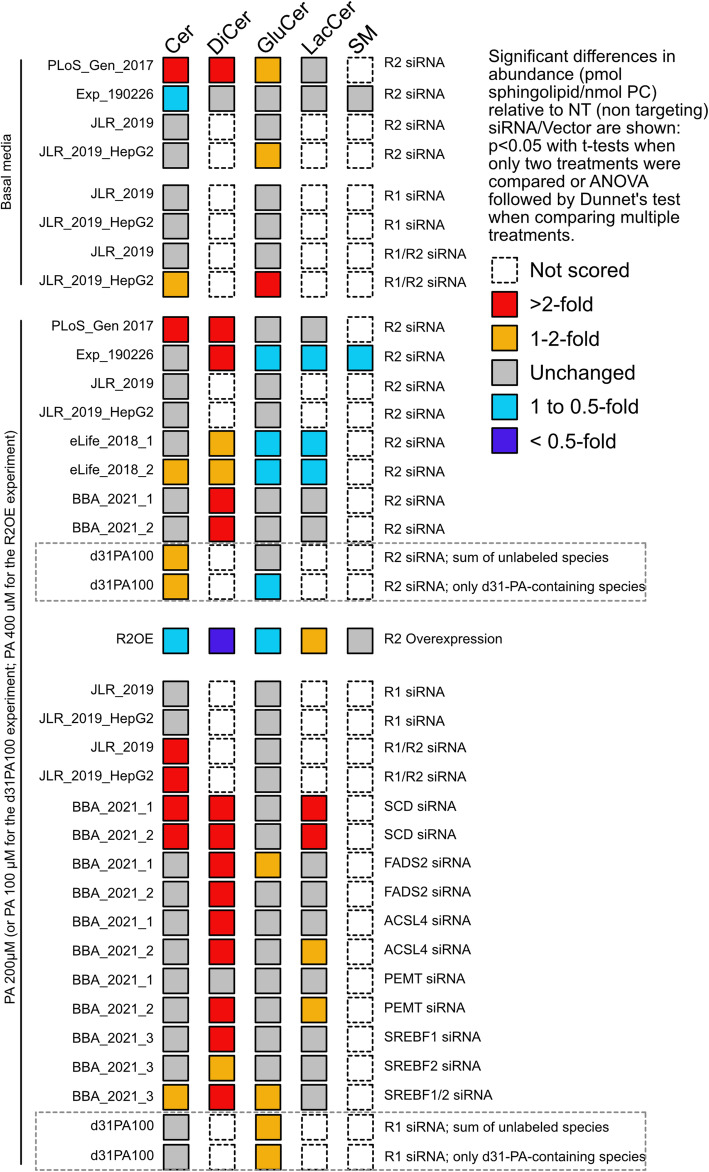
Summary of all experiments. Each row name (left) represents a separate experiment in HEK293 cells (except for JLR_2019_HepG2 that was performed with HepG2 cells) with at least biological triplicates for each condition performed in either basal media (containing BSA only) or in the presence of palmitic acid (conjugated to BSA) and in the presence of the indicated (right) siRNAs or over-expression. Sphingolipids scored were: ceramides (Cer), dihydroceramides (DiCer), glucosylceramides (GluCer), lactosylceramides (LacCer) and sphingomyelins (SM). This sphingolipid data is previously unpublished though some of the experiments were performed as part of published work that included siRNA efficacy assays and phospholipid quantification: PLoS_Gen_2017 [23], JLR_2019 and JLR_2019_HepG2 [29], eLife_2018_1/2 [25], BBA_2021_1/2/3 [30]

A summary of all the experiments is presented in Fig. 2 and shows that lowering AdipoR2 expression consistently leads to increased total dihydroceramide levels when cells are challenged with exogenous PA, while increasing AdipoR2 expression lowers dihydroceramide levels. Furthermore, dihydroceramides are also increased by silencing genes that, like AdipoR2, are important for promoting FA desaturation or the utilization of unsaturated fatty acids (UFA) for phospholipid synthesis, notably the desaturases SCD and FADS2, the acyl-CoA synthase ACSL4 and the transcription factors SREBF1/2. Specific experiments that help clarify the role of the AdipoRs in ceramide homeostasis will now be presented.

### AdipoR2 has a stronger effect than AdipoR1 on sphingolipid homeostasis

**Fig. 3:**
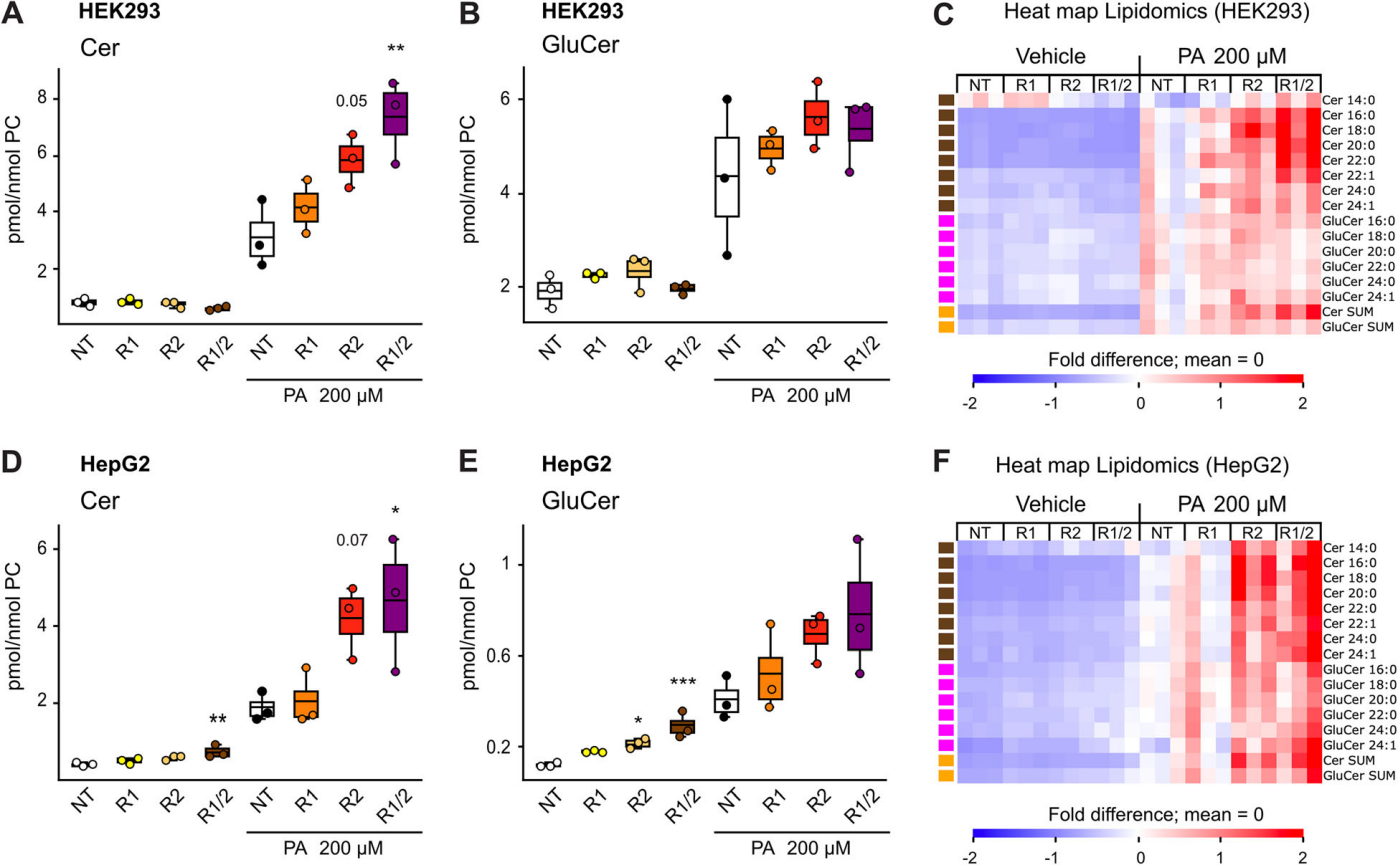
AdipoR2 has a stronger effect than AdipoR1 on sphingolipid levels (data from JLR_2019). The sum of ceramides or glucosylceramides in cells cultured in basal media (containing BSA only) or in the presence of 200 μM palmitic acid (PA; conjugated to BSA) and treated with the indicated siRNAs are shown as box plots while their fatty acid composition is shown as heat maps. **A-C** are results from HEK293 cells and **D-F** from HepG2 cells. In the box plots, boxes indicate the 25th to 75th percentile while the whiskers indicate the data points still within 1.5 of the box range. For **A-D**, significant differences from the NT (non-targeting) siRNA control were determined using one-way ANOVA and post-hoc Dunnett tests, with *P < 0.05, **P < 0.01 and ***P < 0.001. For the heat maps, the amount of each lipid species was normalized to the average of the NT + 200 μM PA treatment and the heat maps show fold differences from the mean across all treatments (each column is a replicate); only lipid species with significantly different levels among siRNAs in a given culture condition are included (ANOVA, q < 0.05)

Fig. 3A-C and Fig. 3D-F show the effects of AdipoR1 and AdipoR2 silencing in HEK293 and HepG2 cells, respectively. Data for only two sphingolipid classes (ceramides and glucosylceramides) were collected in these experiments. Under basal conditions, silencing the AdipoRs had no significant effect in HEK293 cells (Fig. 3A-B). Silencing both AdipoRs caused a small increase in ceramides and glucosylceramides in HepG2 cells, with AdipoR2 silencing having a small effect by itself on glucosylceramide levels (Fig. 3D-E). The addition of PA in the culture media revealed an important role for the AdipoRs in sphingolipid homeostasis. In particular, simultaneously silencing both AdipoR1 and AdipoR2 in HEK293 or HepG2 cells challenged with 200 μM PA caused a significant increase in the total amount of ceramides compared to control cells treated with a NT (non-targeting) siRNA (Fig. 3A and D), with similar effects on species carrying various FAs (Fig. 3C and F). The effect of AdipoR1 silencing on sphingolipids was weaker than that of AdipoR2 silencing, and silencing both genes produced only a small increase in ceramides compared to the effect of AdipoR2 silencing alone when either cell lines is challenged with 200 μM PA. This is consistent with previous studies showing that AdipoR2 is a more potent regulator of membrane homeostasis than AdipoR1 but that the two genes have partially redundant functions [23, 29]. It is for this reason that most of the other experiments will focus on studying the effects of manipulating AdipoR2 levels

### AdipoR2 silencing has little effect on sphingolipids in basal media but increase in dihydroceramides in response to PA

**Fig. 4:**
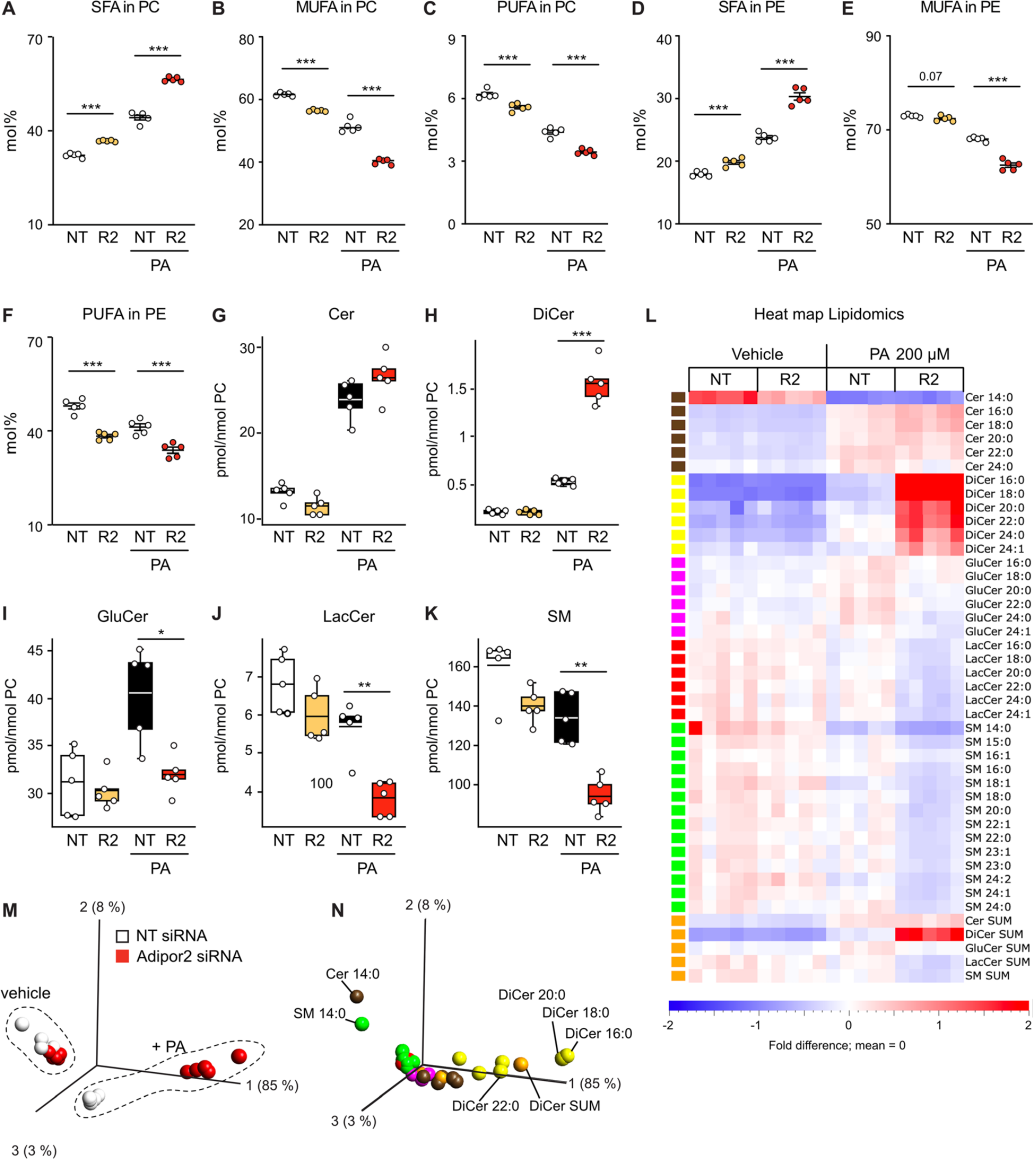
AdipoR2 silencing causes increased dihydroceramide levels (data from Exp_190226). **(A-C)** and **(D-F)** shows the levels of SFA, MUFA and PUFA in phosphatidylcholines (PC) and phosphatidylethanolamines (PE) of HEK293 cells grown in basal media (containing BSA only) or in the presence of 200 μM palmitic acid (PA; conjugated to BSA) and treated with Non-target siRNA (NT) or AdipoR2 siRNA. The sum of five sphingolipid species are as box plots **G-K**) while their fatty acid composition is shown as a heat map **(L)**. **(M)** and **(N)** show principal component analysis plots of the four treatments (vehicle-treated samples are clustered to the left in panel M) and of the lipid species that showed significant differences among treatments (panel N). Note that the strongest effect of AdipoR2 siRNA is the increase in dihydroceramides (see panels H, L and N). In the box plots, boxes indicate the 25th to 75th percentile while the whiskers indicate the data points still within 1.5 of the box range. For A-K, significant differences from the NT siRNA control were determined using Student’s t-tests with *P < 0.05, **P < 0.01 and ***P < 0.001. For the heat map, the amount of each lipid species was normalized to the average of the NT + 200 μM PA treatment and the heat maps show fold differences from the mean across all treatments (each column is a replicate); only lipid species with significantly different levels among siRNAs in a given culture condition are included (ANOVA, q < 0.05)

For the Exp_190226 experiment, data was collected for phosphatidylcholines, phosphatidylethanolamines and five sphingolipid species in HEK293 cells cultivated in basal media or challenged with 200 μM PA. As in previous studies [23, 25, 26, 29, 30], AdipoR2 silencing causes the cells to accumulate excess SFAs at the expense of MUFAs and PUFAs in the phosphatidylcholines and phosphatidylethanolamines under basal conditions and in response to the PA challenge (Fig. 4A-F and Suppl. Fig. S2A-D). This is consistent with AdipoR2 being required for SCD activation, especially in response to a PA membrane-rigidifying challenge.

In the Exp_190266 experiment, AdipoR2 silencing in basal media caused a small decrease in ceramide levels and no significant effect on other sphingolipid classes (Fig. 4G-K). This is consistent with the summary of the results presented in Fig. 2, where AdipoR2 silencing did not have a reproducible effect on any of the sphingolipid species studied when HEK293 (three experiments) or HepG2 (one experiment) were cultivated in basal media. Notably, the ceramide levels increased in AdipoR2 siRNA-treated cells in only one of the four experiments (1/4), were lowered in another (1/4) and unchanged in the remaining two experiment (2/4). This is interesting given that AdipoR2 is a proposed ceramidase: silencing its expression could have resulted in consistently increased ceramide levels, which was not the case.

Inclusion of membrane-rigidifying PA again revealed an important role for AdipoR2 in sphingolipid homeostasis. For example, AdipoR2 silencing in the Exp_190226 experiment caused an increase in the abundance of dihydroceramides and a decrease in lactosylceramides and sphingomyelins in HEK293 cells challenged with 200 μM PA (Fig. 4G-N). The increase in dihydroceramides is the strongest and most reproducible finding from a total of nine sphingolipid quantification (six that include dihydroceramide quantification) experiments in which NT and AdipoR2 siRNA-treated cells were challenged with PA. The results of these experiments are summarized in Fig. 2 and clearly show that increased dihydroceramides is the most reproducible effect of AdipoR2 silencing (6/6 experiments in which they were scored; see Suppl. Fig. S1A-F), and also the strongest effect in terms of fold-increase. The consistent upregulation of dihydroceramides is particularly interesting because they are intermediates in de novo synthesis of ceramides (Fig. 1). Silencing AdipoR2 in the presence of PA did not have a fully reproducible effect on ceramides themselves, although they were moderately increased in 3/9 experiments in which they were measured (8 experiments with HEK293 cells, 1 experiment with HepG2 cells). Cochran’s Q test shows that ceramides (up in 2 of 6 experiments) and dihydroceramides (up in 6 of 6 experiments) were significantly different (*P* = 0.045) in the six experiments where both were measured. Similarly, AdipoR2 silencing led to modest and poorly reproducible reductions in glucosylceramides (reduced in 4/9 experiments), lactosylceramides (reduced in 3/6 experiments) and sphingomyelins (reduced in 1/1 experiment).

In summary, the consistent increase in dihydroceramides in the absence of other reproducible changes suggests that AdipoR2 silencing may induces de novo ceramide synthesis. The alternate hypothesis is that silencing AdipoR2, which has in vitro ceramidase activity, slows down the degradation rate of ceramides and indirectly leads to an accumulation of the dihydroceramides precursors. This alternate hypothesis is less attractive since AdipoR2 silencing did not cause reproducible changes in ceramide levels.

### d31-Labelled PA indicates that AdipoR2 silencing promotes de novo ceramide synthesis

**Fig. 5:**
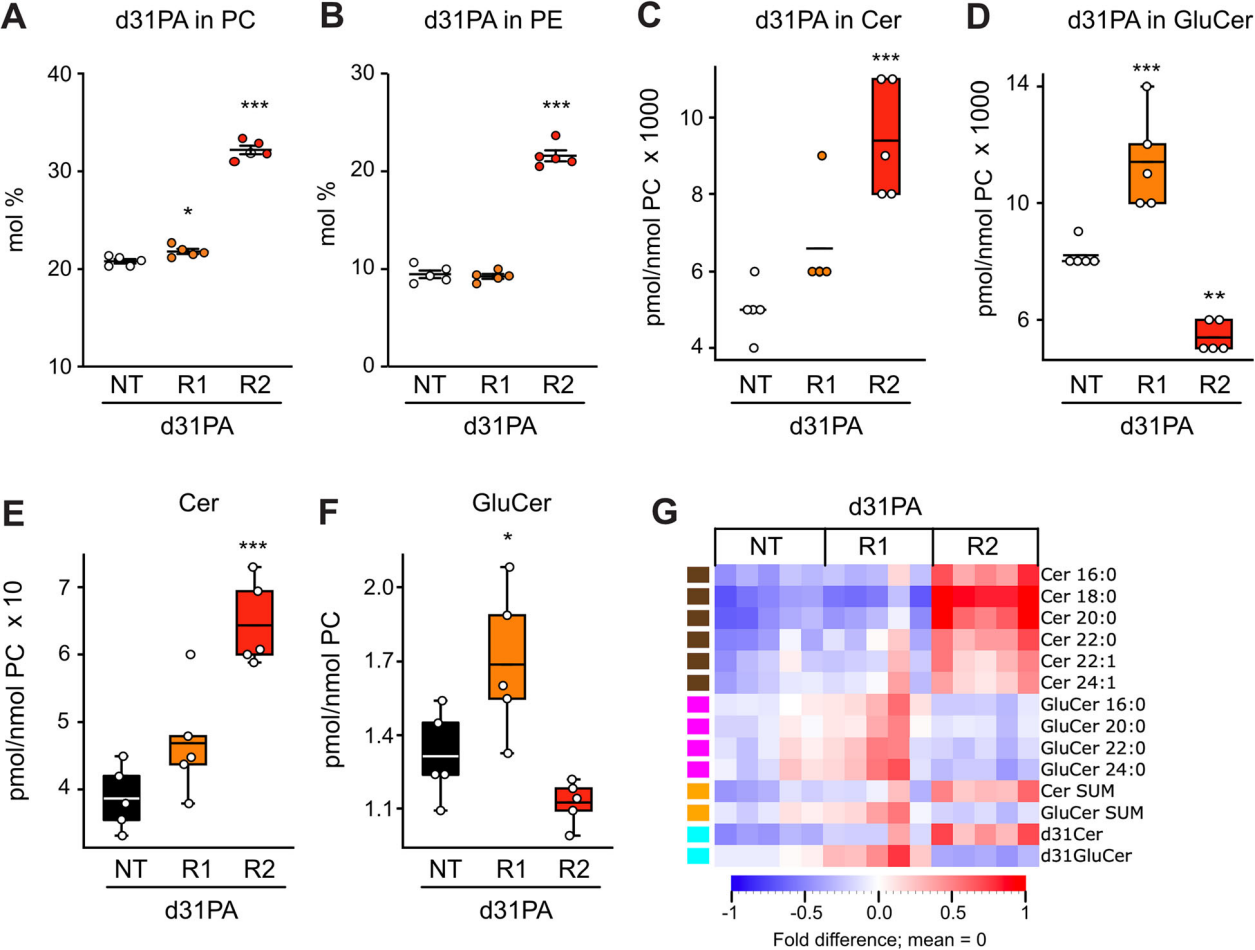
AdipoR2 silencing causes increased incorporation of palmitate into phosphatidylcholines, phosphatidylethanolamines and ceramide (data from d31PA100). (**A**) and (**B**) shows the levels of d31-PA incorporation in phosphatidylcholines (PC) and phosphatidylethanolamines (PE) of HEK293 cells grown in 100 μM d31-PA and treated with Non-target siRNA (NT), AdipoR1 siRNA or AdipoR2 siRNA. The sums of ceramides (Cer) and glucosylceramides (GluCer) species containing d31-PA (**C-D**) or unlabeled fatty acids (**E-F**) are shown as box plots while their fatty acid composition is shown as a heat map (**G**). In the box plots, boxes indicate the 25th to 75th percentile while the whiskers indicate the data points still within 1.5 of the box range. For **A-F**, significant differences from the NT siRNA control were determined using one-way ANOVA and post-hoc Dunnett tests, with *P < 0.05, **P < 0.01 and ***P < 0.001. For the heat map, the amount of each lipid species was normalized to the average of the NT + 100 μM d31PA treatment and the heat maps show fold differences from the mean across all treatments (each column is a replicate); only lipid species with significantly different levels among siRNAs in a given culture condition are included (ANOVA, q < 0.05)

The AdipoRs, and especially AdipoR2, promote fatty acid desaturation in the presence of PA [23, 25, 26, 29, 30], and their silencing could therefore prolong the availability of exogenously supplied PA for de novo ceramide synthesis. To test this hypothesis, NT siRNA-, AdipoR1 siRNA- and AdipoR2 siRNA-treated HEK293 cells were incubated in the presence of 100 μM d31-PA, in which the hydrogen atoms are replaced with deuterium, which allows us to quantify the synthesis of sphingolipids from exogenous PA. As expected, AdipoR2 silencing caused a dramatic increase in the amount of exogenous d31-PA incorporated into both phosphatidylcholines and phosphatidylethanolamines during a 24 h incubation (Fig. 5A-B). Importantly, AdipoR2 silencing also caused increased incorporation of the exogenously supplied d31-PA into new ceramides (Cer d31–16:0; Fig. 5C) and decreased incorporation into new glucosylceramides (GluCer d31–16:0; Fig. 5D). This suggests that AdipoR2 activity normally decreases de novo ceramide synthesis from exogenously supplied PA. Interestingly, the increase in d31-labeled ceramides and decrease in d31-labeled glucosylceramides in AdipoR2 siRNA-treated cells were mirrored among the unlabeled species (Fig. 5E-F). This suggests that AdipoR2 silencing can promote increased ceramide containing both the exogenously supplied 31d-PA (hence through de novo synthesis) as well as endogenous unlabeled PA, which may itself be de novo synthesized but not desaturated efficiently when AdipoR2 is silenced.

AdipoR1 silencing had no significant effect on the levels of ceramide levels (Fig. 5C, E, G) but caused an increase in the levels of glucosylceramides (Fig. 5D, F-G). AdipoR1 and AdipoR2 may therefore have different effects on sphingolipids.

### Overexpression of AdipoR2 causes decreased ceramides and dihydroceramides

**Fig. 6:**
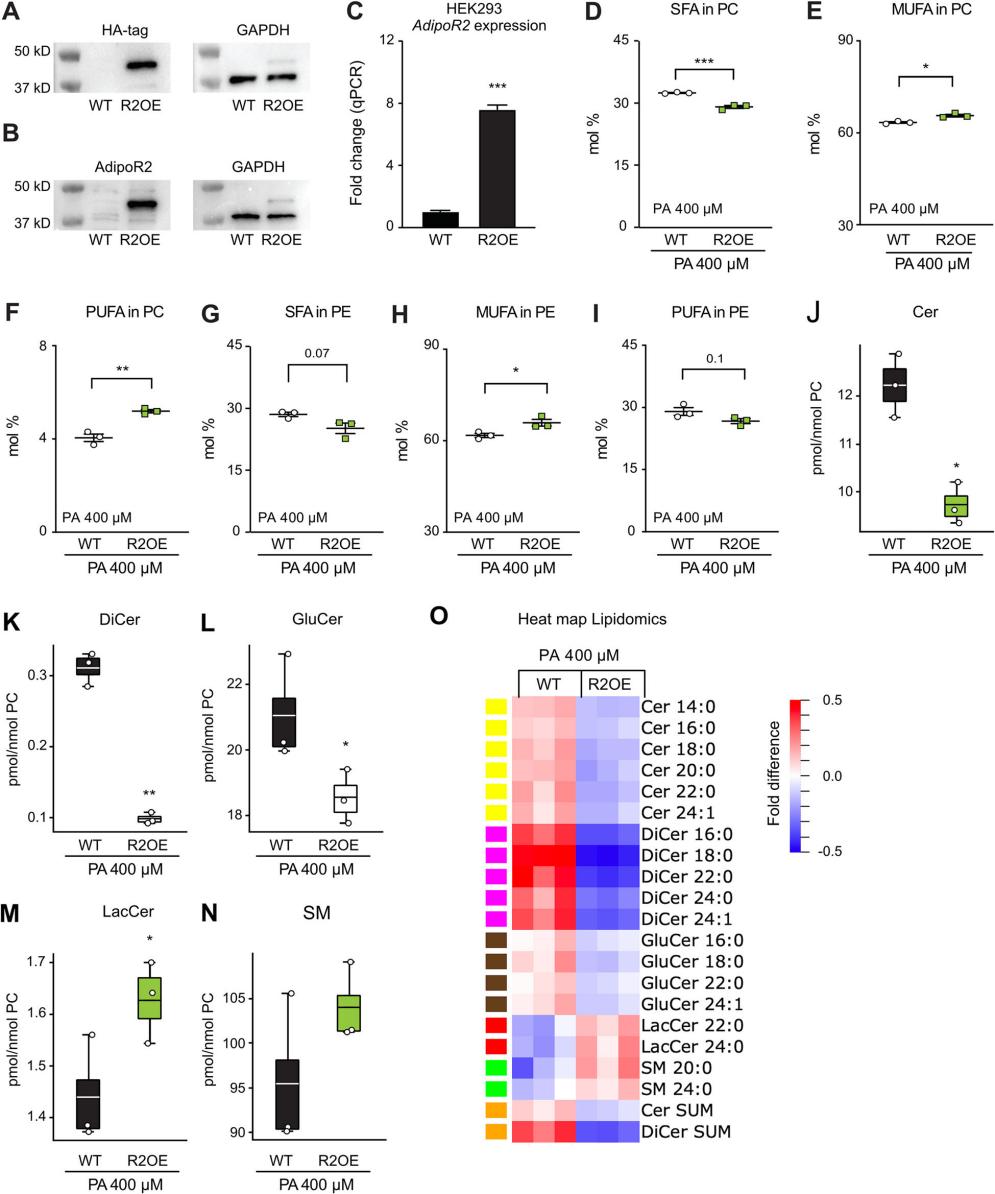
AdipoR2 overexpression causes decreased dihydroceramide levels (data from the R2OE experiment). Western blots detection of the HA-tagged over-expressed AdipoR2 using an anti-HA antibody (**A**) or an anti-AdipoR2 antibody (**B**); anti-GAPDH was used as a loading control. **(D-F)** Relative abundance of SFA, MUFA and PUFA in phosphatidylcholines (PC) and phosphatidylethanolamines (PE) of control (WT) and AdipoR2 over-expressing cells (R2OE) treated with 400 μM PA for 6 h. The sums of five sphingolipid classes are shown as box plots (**J-N**) while their fatty acid composition is shown as a heat map (**O**). In the box plots, boxes indicate the 25th to 75th percentile while the whiskers indicate the data points still within 1.5 of the box range. For D-N, significant differences from the WT control were determined using Student’s t-tests, with *P < 0.05, **P < 0.01 and ***P < 0.001. For the heat map, the amount of each lipid species was normalized to the average of the WT + 400 μM PA treatment and the heat maps show fold differences from the mean across all treatments (each column is a replicate); only lipid species with significantly different levels among siRNAs in a given culture condition are included (ANOVA, q < 0.05)

A HEK293 line was created that stably overexpresses AdipoR2 both at the protein (Fig. 6A-B) and mRNA level (Fig. 6C). The over-expression of AdipoR2 caused a decreased abundance of SFAs accompanied by increased MUFAs in both phosphatidylcholines and phosphatidylethanolamines (Fig. 6D-E and G-H), and increased levels of PUFAs in the phophatidylcholines (Fig. 6F) but not in phosphatidylethanolamines (Fig. 6I). This again is consistent with AdipoR2 acting to promote fatty acid desaturation [23, 25, 26, 29, 30]. Previous studies showed that transient AdipoR2 over-expression protects against membrane rigidification when cells are challenged with a high PA dose (400 μM) for 6 h, which is sufficient to causes rigidification in control cells [27]. Here, cells stably over-expressing AdipoR2 were also protected from the effects that a high PA dose (400 μM, 6 h) has on ceramides and dihydroceramides: the AdipoR2 overexpressing cells showed a ~ 20% decrease in ceramides (from 13.25 to 10.75 pmol/nmol PC) and a 67% decrease in dihydroceramides (from 0.31 to 0.1 pmol/nmol PC) (Fig. 6J-K). Additionally, all ceramide and dihydroceramide species were equally affected (Fig. 6O), and only small changes were found in total glucosylceramides, lactosylceramides or sphingomyelins (Fig. 6L-N). In conclusion, overexpression of AdipoR2 has its clearest effect on dihydroceramides, which is again consistent with AdipoR2 activity causing reduced ceramide de novo ceramide synthesis.

### Inhibition of desaturases is a very potent way to increase sphingolipid levels

**Fig. 7:**
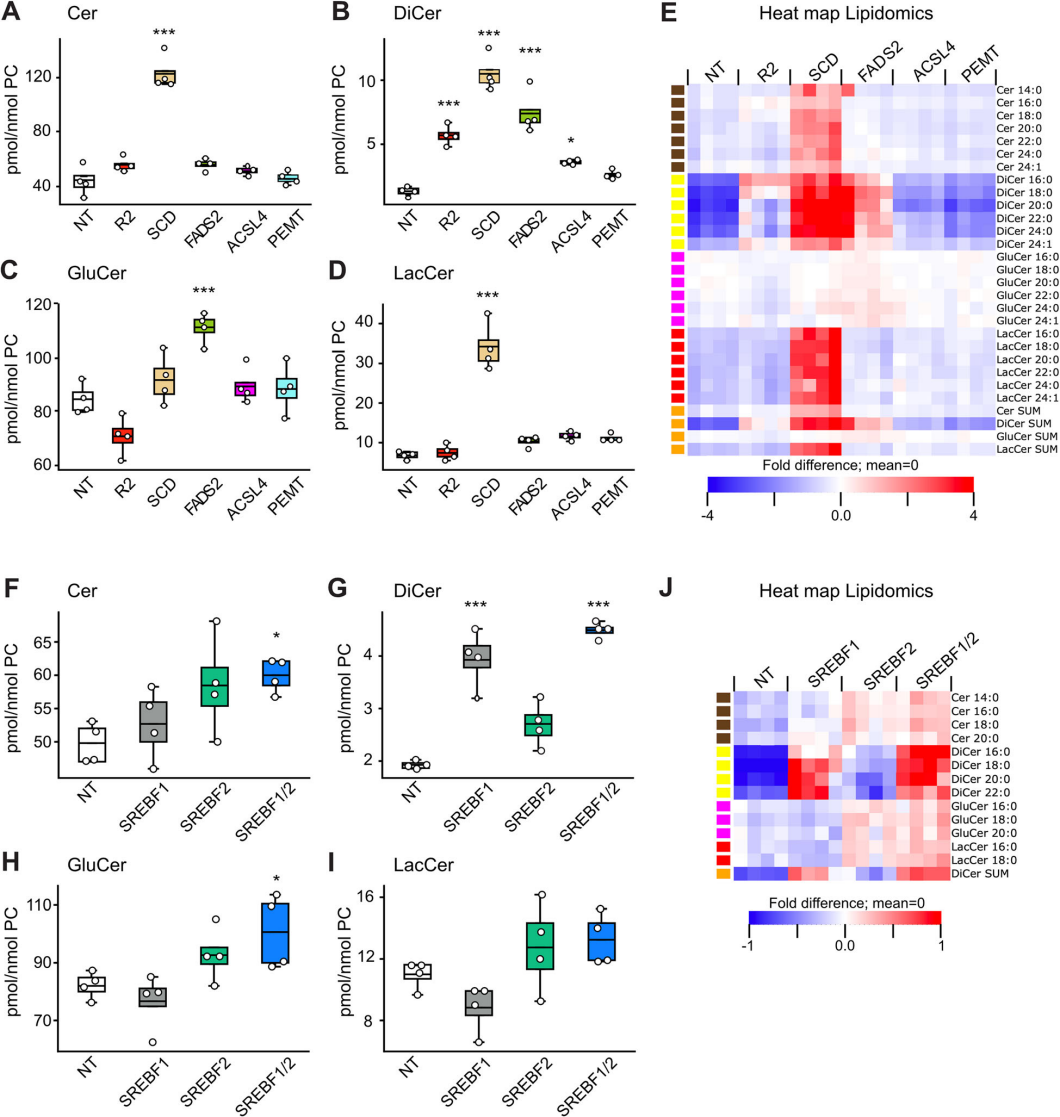
Desaturase or SREBF silencing causes elevated dihydroceramide levels (data from BBA_2021_1 and BBA_2021_3). The sum of four sphingolipid species in HEK293 cells treated with the indicated siRNAs then cultivated in the presence of 200 μM PA are shown as box plots while their fatty acid composition is shown as heat maps. In the box plots, boxes indicate the 25th to 75th percentile while the whiskers indicate the data points still within 1.5 of the box range. For **A-D** and **F-I,** significant differences from the NT siRNA control were determined using one-way ANOVA and post-hoc Dunnett tests, with *P < 0.05, **P < 0.01 and ***P < 0.001. For the heat maps, the amount of each lipid species was normalized to the average of the NT + 200 μM PA treatment and the heat maps show fold differences from the mean across all treatments (each column is a replicate); only lipid species with significantly different levels among siRNAs in a given culture condition are included (ANOVA, q < 0.05)

As already mentioned, previous studies on AdipoR2 have shown that it stimulates desaturase expression in response to membrane-rigidifying PA [23, 29, 30]. If the effect of AdipoR2 on dihydroceramides is mediated by its effect on desaturase expression, then direct inhibition of genes important for fatty acid desaturation should also lead to increased dihydroceramide levels. Indeed, siRNA inhibition of the desaturase SCD was more potent than AdipoR2 silencing in causing increased dihydroceramide levels (SCD siRNA caused a near 10-fold dihydroceramide increase compared to a ~ 4-fold increase with AdipoR2 siRNA; Fig. 7A-E). Silencing three other genes previously implicated in resistance to PA [30, 50, 51], namely FADS2 (a desaturase), ACSL4 (an acyl-CoA transferase that channels UFAs towards phospholipid incorporation), and PEMT (an enzyme that converts PEs into PCs), also caused increased dihydroceramide levels, though not as potently as SCD silencing (Fig. 7A-E). Previous work showed that the transcriptome of cells lacking AdipoR2 and challenged with PA likens that of cells in which the membrane homeostasis SREBP pathway is silenced, and specifically that AdipoR2 silencing led to under-expression of INSIG1, SREBF2 and several downstream desaturases, including SCD, SCD5, FADS1 and FADS2 [30]. The RNAseq data for the genes here studied is presented in Suppl. Fig. S3 and shows that the expression of FADS2, PEMT, SCD and SREBF2 is reduced in AdipoR2-KO cells (Suppl. Fig. S3). Here, silencing of SREBF1 and SREBF2 also caused a ~ 3-fold dihydroceramide increase, i.e. levels similar to those observed when AdipoR2 is silenced (Fig. 7F-J). It is interesting to note that SCD also caused a strong increase in ceramides and lactosylceramides (Fig. 7A, D-E), suggesting that it has a broader effect on sphingolipid homeostasis than AdipoR2, which is consistent with SCD being a downstream target of multiple pathways.

## Discussion

The present study monitored the abundance and composition of several sphingolipid classes in response to various treatments, notably silencing or overexpression of AdipoRs and other genes. The main findings of the present study are that AdipoR2 silencing predominantly results in elevated dihydroceramide levels (reproduced in 6/6 experiments) and that this effect can also be achieved by silencing fatty acid desaturases. Dihydroceramides are generated during de novo ceramide synthesis (Fig. 1). A plausible interpretation of our findings is therefore that silencing the AdipoRs, and especially AdipoR2, results in decreased desaturase expression that causes elevated levels of intracellular PA, which in turn increases de novo ceramide synthesis via the rate-limiting step catalyzed by serine palmitoyltransferase. This interpretation is in line with the earlier observation that the AdipoRs, and especially AdipoR2, are required for the upregulation of desaturases (SCD, FADS1 and FADS2) in the presence of membrane-rigidifying PA [12, 23–26, 28–30].

The membrane homeostasis function of the AdipoRs is evolutionarily conserved in their *C. elegans* and yeast counterparts [12]. Additionally, there is considerable evidence that the AdipoRs and their distant yeast homolog IZH2 are hydrolases, and specifically act as ceramidases. In particular, IZH2 shows sequence homology with ceramidases, signals via sphingoid bases (a product of the ceramidase reaction) and its pathway is inhibited by myriocin, a serine palmitoyltransferase inhibitor [36]. Also, the human AdipoR1 and AdipoR2 proteins can partially functionally replace IZH2 in yeast [34], and the recombinant AdipoRs exhibit a ceramidase activity in vitro, albeit at the extremely low rate of 0.03 reactions per site per minute [33]. In summary, it is likely that the AdipoRs have some ceramidase activity, though it remains unclear whether they may have preference for specific ceramide species or even other types of substrates [52]. In the experiments described here, there was no reproducible increase in ceramide levels when AdipoR2 was silenced (though there was a tendency in that direction since ceramides were significantly elevated in 3/9 experiments in PA-challenged cells; Fig. 2). These results suggest that any ceramidase activity that the AdipoRs may have does not always measurably impact bulk ceramide levels, though it may be sufficient to generate important signaling molecules such as sphingosine, as previously suggested [38].

Transgenic mice overexpressing the AdipoRs show reduced ceramide levels in liver and fat depots [37] while mouse embryonic fibroblasts lacking the AdipoRs show elevated ceramide levels [38]. These findings are consistent with the AdipoRs having an in vivo ceramidase activity potent enough to affect ceramide level in cells and throughout entire organs. However, the present study suggests a complementary explanation, namely that modulating AdipoR expression mostly affects ceramide levels indirectly by influencing desaturase expression, hence availability of the rate-limiting intracellular PA. Importantly, this complicates the interpretation of the lowered/increased ceramide levels in AdipoR overexpressing/deficient mouse models since any change in ceramide levels would also be accompanied by corresponding changes in SFA levels. Numerous studies have implicated both ceramides and SFAs as contributors to impaired insulin signaling and metabolic complications. Dihydroceramides, the levels of which were most consistently increased in response to lowered AdipoR2 activity, are now emerging as having important physiological roles including promoting autophagy [53–55] and inhibiting adipogenesis [56, 57]. Further, elevated plasma dihydroceramides appears to be the only lipid value that is consistently predictive of diabetes for up to 9 years before its onset [58]. In the future, it will therefore be important to try and dissect the respective roles of SFAs, dihydroceramides and ceramides in the AdipoR transgenic/knockout mouse models and human studies.

### Study strengths

Sphingolipids were quantified across numerous experiments and conditions, which allowed for the identification of the most reproducible consequences of silencing AdipoR2 and other genes on sphingolipid homeostasis.

### Limitations

Most experiments were carried out with one cell line, namely HEK293. However, previous studies have shown that HEK293 is a representative and useful model to study the roles of the AdipoRs in membrane homeostasis since findings in that cell line are reproducible in a variety of other cell types/models [29, 30].

## Conclusions

In summary, the AdipoRs are clearly implicated in sphingolipid homeostasis and the most reproducible consequence of silencing AdipoR2 is an increase in dihydroceramides. This is consistent with AdipoR2 activity causing reduced de novo ceramide synthesis. Mechanistically, AdipoR2 silencing likely leads to increased intracellular supply of palmitic acid that in turn leads to increased dihydroceramide synthesis via the rate-limiting serine palmitoyl transferase step. In agreement with this model, inhibiting the desaturase SCD or SREBF1/2 (positive regulators of SCD) also causes a strong increase in dihydroceramide levels. Knowing that the AdipoRs act primarily by promoting fatty acid desaturation to maintain membrane fluidity, and that their effect on dihydroceramides and ceramides is secondary, will help guide clinical studies. Speculatively, patients with elevated membrane SFAs accompanied by elevated dihydroceramide levels may be indicative of AdipoR signaling deficiency that could be treated by AdipoR agonists or SCD activators.

## Supplementary Information

**Additional file 1.**

## Data Availability

Data and materials would be supplied on reasonable requests.

## Abbreviations

AdipoR: Adiponectin receptor
ATCC: American Type Culture Collection
BSA: Bovine serum albumin
MUFA: Monounsaturated fatty acid
NT siRNA: Non targeting siRNA
PA: Palmitic acid
PAQR: Progestin and adipoQ receptor
PC: phosphatidylcholine
qPCR: quantitative polymerase chain reaction
SFA: Saturated fatty acid
UFA: Unsaturated fatty acid

## References

[1] Alonso A, Goni FM. The physical properties of ceramides in membranes. Annu Rev Biophys. 2018;47:633–54. 10.1146/annurev-biophys-070317-033309 29618220

[2] Kolesnick R (2002). The therapeutic potential of modulating the ceramide/sphingomyelin pathway. J Clin Invest.

[3] Summers SA, Garza LA, Zhou H, Birnbaum MJ (1998). Regulation of insulin-stimulated glucose transporter GLUT4 translocation and Akt kinase activity by ceramide. Mol Cell Biol.

[4] Chavez JA, Knotts TA, Wang LP, Li G, Dobrowsky RT, Florant GL, Summers SA (2003). A role for ceramide, but not diacylglycerol, in the antagonism of insulin signal transduction by saturated fatty acids. J Biol Chem.

[5] Chavez JA, Holland WL, Bar J, Sandhoff K, Summers SA (2005). Acid ceramidase overexpression prevents the inhibitory effects of saturated fatty acids on insulin signaling. J Biol Chem.

[6] Turpin SM, Lancaster GI, Darby I, Febbraio MA, Watt MJ (2006). Apoptosis in skeletal muscle myotubes is induced by ceramides and is positively related to insulin resistance. Am J Physiol Endocrinol Metab.

[7] Xia JY, Holland WL, Kusminski CM, Sun K, Sharma AX, Pearson MJ, et al. Targeted induction of ceramide degradation leads to improved systemic metabolism and reduced hepatic steatosis. Cell Metab. 2015;22:266–78. 10.1016/j.cmet.2015.06.007 PMC4527941 26190650

[8] Poss AM, Holland WL, Summers SA (2020). Risky lipids: refining the ceramide score that measures cardiovascular health. Eur Heart J.

[9] Tippetts TS, Holland WL, Summers SA (2018). The ceramide ratio: a predictor of cardiometabolic risk. J Lipid Res.

[10] Listenberger LL, Ory DS, Schaffer JE (2001). Palmitate-induced apoptosis can occur through a ceramide-independent pathway. J Biol Chem.

[11] Wei Y, Wang D, Topczewski F, Pagliassotti MJ (2006). Saturated fatty acids induce endoplasmic reticulum stress and apoptosis independently of ceramide in liver cells. Am J Physiol Endocrinol Metab.

[12] Pilon M (2021). Paradigm shift: the primary function of the "Adiponectin receptors" is to regulate cell membrane composition. Lipids Health Dis.

[13] Tang YT, Hu T, Arterburn M, Boyle B, Bright JM, Emtage PC, Funk WD (2005). PAQR proteins: a novel membrane receptor family defined by an ancient 7-transmembrane pass motif. J Mol Evol.

[14] Yamauchi T, Kamon J, Ito Y, Tsuchida A, Yokomizo T, Kita S, Sugiyama T, Miyagishi M, Hara K, Tsunoda M (2003). Cloning of adiponectin receptors that mediate antidiabetic metabolic effects. Nature.

[15] Degreif D, de Rond T, Bertl A, Keasling JD, Budin I (2017). Lipid engineering reveals regulatory roles for membrane fluidity in yeast flocculation and oxygen-limited growth. Metab Eng.

[16] Karpichev IV, Cornivelli L, Small GM. Multiple regulatory roles of a novel *Saccharomyces cerevisiae* protein, encoded by YOL002c, in lipid and phosphate metabolism. J Biol Chem. 2002;277:19609–17. 10.1074/jbc.M202045200 11916977

[17] Karpichev IV, Small GM. Global regulatory functions of Oaf1p and Pip2p (Oaf2p), transcription factors that regulate genes encoding peroxisomal proteins in *Saccharomyces cerevisiae*. Mol Cell Biol. 1998;18:6560–70. 10.1128/mcb.18.11.6560 PMC109241 9774671

[18] Lyons TJ, Villa NY, Regalla LM, Kupchak BR, Vagstad A, Eide DJ (2004). Metalloregulation of yeast membrane steroid receptor homologs. Proc Natl Acad Sci U S A.

[19] Mattiazzi Usaj M, Prelec M, Brloznik M, Primo C, Curk T, Scancar J, et al. Yeast Saccharomyces cerevisiae adiponectin receptor homolog Izh2 is involved in the regulation of zinc, phospholipid and pH homeostasis. Metallomics. 2015;7:1338–51. 10.1039/c5mt00095e 26067383

[20] Svensson E, Olsen L, Morck C, Brackmann C, Enejder A, Faergeman NJ, Pilon M: The adiponectin receptor homologs in *C. elegans* promote energy utilization and homeostasis. PLoS One. 2011;6:e21343. 10.1371/journal.pone.0021343 PMC3119701 21712952

[21] Svensk E, Stahlman M, Andersson CH, Johansson M, Boren J, Pilon M (2013). PAQR-2 regulates fatty acid desaturation during cold adaptation in *C. elegans*. PLoS Genet.

[22] Svensk E, Devkota R, Stahlman M, Ranji P, Rauthan M, Magnusson F, et al. *Caenorhabditis elegans* PAQR-2 and IGLR-2 protect against glucose toxicity by modulating membrane lipid composition. PLoS Genet. 2016;12:e1005982. 10.1371/journal.pgen.1005982 PMC4833288 27082444

[23] Devkota R, Svensk E, Ruiz M, Stahlman M, Boren J, Pilon M. The adiponectin receptor AdipoR2 and its *Caenorhabditis elegans* homolog PAQR-2 prevent membrane rigidification by exogenous saturated fatty acids. PLoS Genet. 2017;13:e1007004. 10.1371/journal.pgen.1007004 PMC5607217 28886012

[24] Bodhicharla R, Devkota R, Ruiz M, Pilon M. Membrane fluidity is regulated cell nonautonomously by *Caenorhabditis elegans* PAQR-2 and its mammalian homolog AdipoR2. Genetics. 2018;210:189–201. 10.1534/genetics.118.301272 PMC6116961 29997234

[25] Ruiz M, Bodhicharla R, Svensk E, Devkota R, Busayavalasa K, Palmgren H, Stahlman M, Boren J, Pilon M: Membrane fluidity is regulated by the *C. elegans* transmembrane protein FLD-1 and its human homologs TLCD1/2. Elife 2018, 7:e40686. 10.7554/eLife.40686 PMC6279351 30509349

[26] Ruiz M, Bodhicharla R, Stahlman M, Svensk E, Busayavalasa K, Palmgren H, Ruhanen H, Boren J, Pilon M (2019). Evolutionarily conserved long-chain acyl-CoA synthetases regulate membrane composition and fluidity. Elife.

[27] Busayavalasa K, Ruiz M, Devkota R, Stahlman M, Bodhicharla R, Svensk E, et al. Leveraging a gain-of-function allele of *Caenorhabditis elegans paqr-1* to elucidate membrane homeostasis by PAQR proteins. PLoS Genet. 2020;16:e1008975. 10.1371/journal.pgen.1008975 PMC7428288 32750056

[28] Devkota R, Henricsson M, Boren J, Pilon M: The *C. elegans* PAQR-2 and IGLR-2 membrane homeostasis proteins are uniquely essential for tolerating dietary saturated fats. Biochim Biophys Acta Mol Cell Biol Lipids 2021, 1866:158883. 10.1016/j.bbalip.2021.158883 33444761

[29] Ruiz M, Stahlman M, Boren J, Pilon M (2019). AdipoR1 and AdipoR2 maintain membrane fluidity in Most human cell types and independently of Adiponectin. J Lipid Res.

[30] Ruiz M, Palmgren H, Henricsson M, Devkota R, Jaiswal H, Maresca M, Bohlooly YM, Peng XR, Boren J, Pilon M (1866). Extensive transcription mis-regulation and membrane defects in AdipoR2-deficient cells challenged with saturated fatty acids. Biochim Biophys Acta Mol Cell Biol Lipids.

[31] Pei J, Millay DP, Olson EN, Grishin NV (2011). CREST--a large and diverse superfamily of putative transmembrane hydrolases. Biol Direct.

[32] Tanabe H, Fujii Y, Okada-Iwabu M, Iwabu M, Nakamura Y, Hosaka T, Motoyama K, Ikeda M, Wakiyama M, Terada T (2015). Crystal structures of the human adiponectin receptors. Nature.

[33] Vasiliauskaite-Brooks I, Sounier R, Rochaix P, Bellot G, Fortier M, Hoh F, De Colibus L, Bechara C, Saied EM, Arenz C (2017). Structural insights into adiponectin receptors suggest ceramidase activity. Nature.

[34] Kupchak BR, Garitaonandia I, Villa NY, Mullen MB, Weaver MG, Regalla LM, Kendall EA, Lyons TJ (1773). Probing the mechanism of FET3 repression by Izh2p overexpression. Biochim Biophys Acta.

[35] Kupchak BR, Garitaonandia I, Villa NY, Smith JL, Lyons TJ (2009). Antagonism of human adiponectin receptors and their membrane progesterone receptor paralogs by TNFalpha and a ceramidase inhibitor. Biochemistry.

[36] Villa NY, Kupchak BR, Garitaonandia I, Smith JL, Alonso E, Alford C, Cowart LA, Hannun YA, Lyons TJ (2009). Sphingolipids function as downstream effectors of a fungal PAQR. Mol Pharmacol.

[37] Holland WL, Xia JY, Johnson JA, Sun K, Pearson MJ, Sharma AX, Quittner-Strom E, Tippetts TS, Gordillo R, Scherer PE (2017). Inducible overexpression of adiponectin receptors highlight the roles of adiponectin-induced ceramidase signaling in lipid and glucose homeostasis. Mol Metab.

[38] Holland WL, Miller RA, Wang ZV, Sun K, Barth BM, Bui HH, Davis KE, Bikman BT, Halberg N, Rutkowski JM (2011). Receptor-mediated activation of ceramidase activity initiates the pleiotropic actions of adiponectin. Nat Med.

[39] Hanada K, Hara T, Nishijima M (2000). Purification of the serine palmitoyltransferase complex responsible for sphingoid base synthesis by using affinity peptide chromatography techniques. J Biol Chem.

[40] Mandon EC, Ehses I, Rother J, van Echten G, Sandhoff K (1992). Subcellular localization and membrane topology of serine palmitoyltransferase, 3-dehydrosphinganine reductase, and sphinganine N-acyltransferase in mouse liver. J Biol Chem.

[41] Merrill AH (1983). Characterization of serine palmitoyltransferase activity in Chinese hamster ovary cells. Biochim Biophys Acta.

[42] Williams RD, Wang E, Merrill AH (1984). Enzymology of long-chain base synthesis by liver: characterization of serine palmitoyltransferase in rat liver microsomes. Arch Biochem Biophys.

[43] Livak KJ, Schmittgen TD (2001). Analysis of relative gene expression data using real-time quantitative PCR and the 2(−Delta Delta C(T)) method. Methods.

[44] Bjursell M, Ahnmark A, Bohlooly YM, William-Olsson L, Rhedin M, Peng XR, Ploj K, Gerdin AK, Arnerup G, Elmgren A (2007). Opposing effects of adiponectin receptors 1 and 2 on energy metabolism. Diabetes.

[45] Lofgren L, Forsberg GB, Stahlman M (2016). The BUME method: a new rapid and simple chloroform-free method for total lipid extraction of animal tissue. Sci Rep.

[46] Jung HR, Sylvanne T, Koistinen KM, Tarasov K, Kauhanen D, Ekroos K (1811). High throughput quantitative molecular lipidomics. Biochim Biophys Acta.

[47] Ejsing CS, Sampaio JL, Surendranath V, Duchoslav E, Ekroos K, Klemm RW, Simons K, Shevchenko A (2009). Global analysis of the yeast lipidome by quantitative shotgun mass spectrometry. Proc Natl Acad Sci U S A.

[48] Ekroos K, Ejsing CS, Bahr U, Karas M, Simons K, Shevchenko A (2003). Charting molecular composition of phosphatidylcholines by fatty acid scanning and ion trap MS3 fragmentation. J Lipid Res.

[49] Amrutkar M, Cansby E, Nunez-Duran E, Pirazzi C, Stahlman M, Stenfeldt E, Smith U, Boren J, Mahlapuu M (2015). Protein kinase STK25 regulates hepatic lipid partitioning and progression of liver steatosis and NASH. FASEB J.

[50] Piccolis M, Bond LM, Kampmann M, Pulimeno P, Chitraju C, Jayson CBK, Vaites LP, Boland S, Lai ZW, Gabriel KR (2019). Probing the global cellular responses to lipotoxicity caused by saturated fatty acids. Mol Cell.

[51] Zhu XG, Puthenveedu SN, Shen YH, La K, Ozlu C, Wang T, et al. CHP1 regulates compartmentalized glycerolipid synthesis by activating GPAT4. Mol Cell. 2019;74:45–58. 10.1016/j.molcel.2019.01.037 PMC6450717 30846317

[52] Muratore M, Komai AM (2020). Theoretical study of the adiponectin receptors: binding site characterization and molecular dynamics of possible ligands for drug design. SN Applied Sciences.

[53] Gagliostro V, Casas J, Caretti A, Abad JL, Tagliavacca L, Ghidoni R, Fabrias G, Signorelli P (2012). Dihydroceramide delays cell cycle G1/S transition via activation of ER stress and induction of autophagy. Int J Biochem Cell Biol.

[54] Signorelli P, Munoz-Olaya JM, Gagliostro V, Casas J, Ghidoni R, Fabrias G (2009). Dihydroceramide intracellular increase in response to resveratrol treatment mediates autophagy in gastric cancer cells. Cancer Lett.

[55] Zheng W, Kollmeyer J, Symolon H, Momin A, Munter E, Wang E, Kelly S, Allegood JC, Liu Y, Peng Q (1758). Ceramides and other bioactive sphingolipid backbones in health and disease: lipidomic analysis, metabolism and roles in membrane structure, dynamics, signaling and autophagy. Biochim Biophys Acta.

[56] Barbarroja N, Rodriguez-Cuenca S, Nygren H, Camargo A, Pirraco A, Relat J, Cuadrado I, Pellegrinelli V, Medina-Gomez G, Lopez-Pedrera C (2015). Increased dihydroceramide/ceramide ratio mediated by defective expression of degs1 impairs adipocyte differentiation and function. Diabetes.

[57] Lachkar F, Ferre P, Foufelle F, Papaioannou A (2021). Dihydroceramides: their emerging physiological roles and functions in cancer and metabolic diseases. Am J Physiol Endocrinol Metab.

[58] Wigger L, Cruciani-Guglielmacci C, Nicolas A, Denom J, Fernandez N, Fumeron F, et al. Plasma dihydroceramides are diabetes susceptibility biomarker candidates in mice and humans. Cell Rep. 2017;18:2269–79. 10.1016/j.celrep.2017.02.019 28249170

