# Supplementary material for: Palmitic acid causes increased dihydroceramide levels when desaturase expression is directly silenced or indirectly lowered by silencing AdipoR2
Source: Lipids Health Dis. 2021 Nov 28;20:173. doi: 10.1186/s12944-021-01600-y (PMC8627610; doi:10.1186/s12944-021-01600-y)
Supplement: Supplementary file 1 — Additional file 1. [file 12944_2021_1600_MOESM1_ESM.pdf]

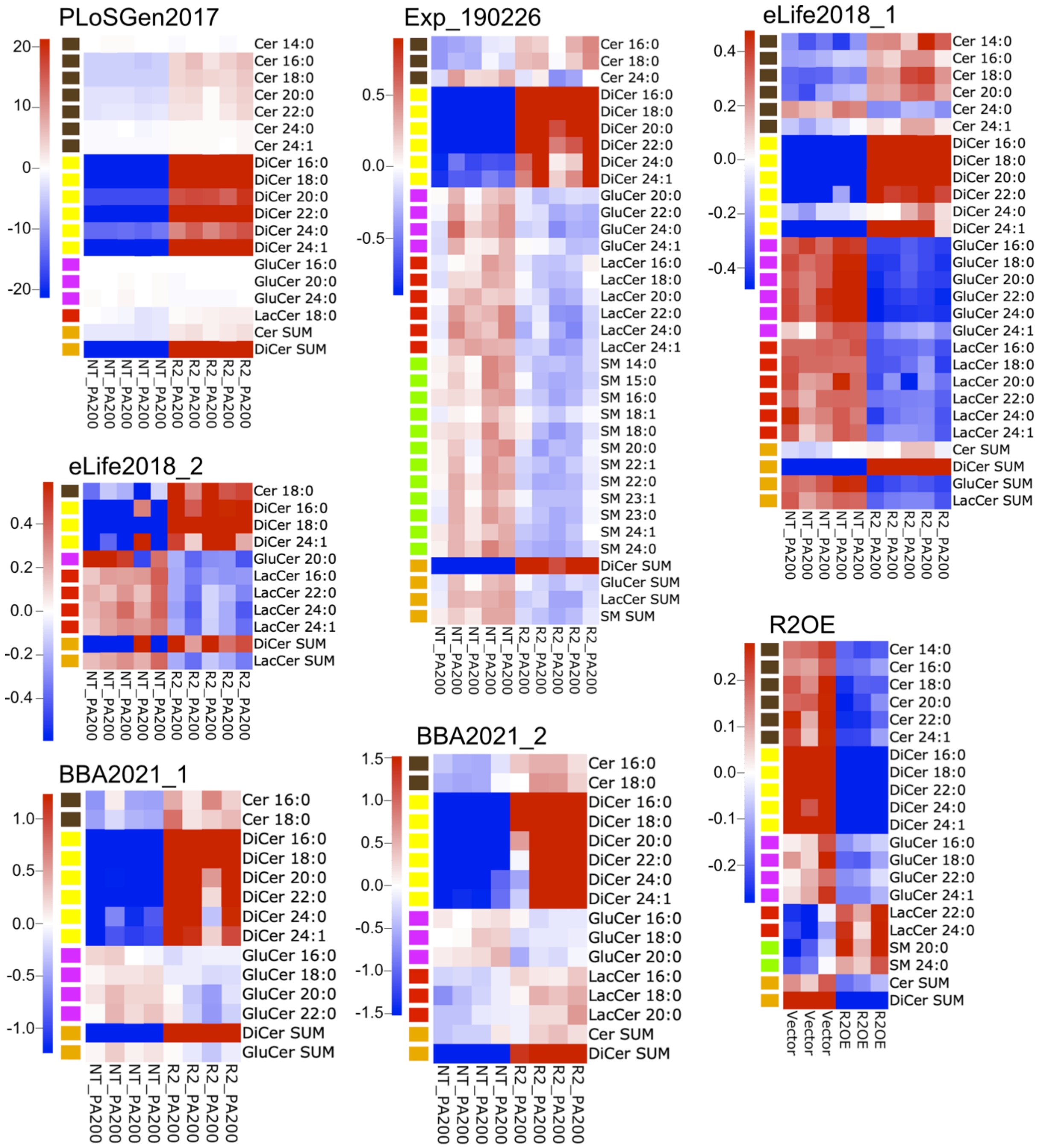

Suppl. Fig. S1. Dihydroceramides are the sphingolipids most affected by changes in AdipoR2 expression. All experiments that included quantification of dihydroceramides are shown here. Default scales provided by the Qlucore data analysis software were used, which tend to oversaturate but provide strong contrast. The heat maps strikingly show that the most consistent and pronounced change across all experiments is the strong increase in dihydroceramide when ADIPOR2 is silenced (**A-F**). Conversely, dihydroceramides are also the most decreased lipid species when ADIPOR2 is overexpressed (**G**). Only lipid species that significantly differed between the two treatments are shown (stat: two-group comparison, $P<0.05$). Scale bars show fold difference from the mean of all samples for each lipid species.

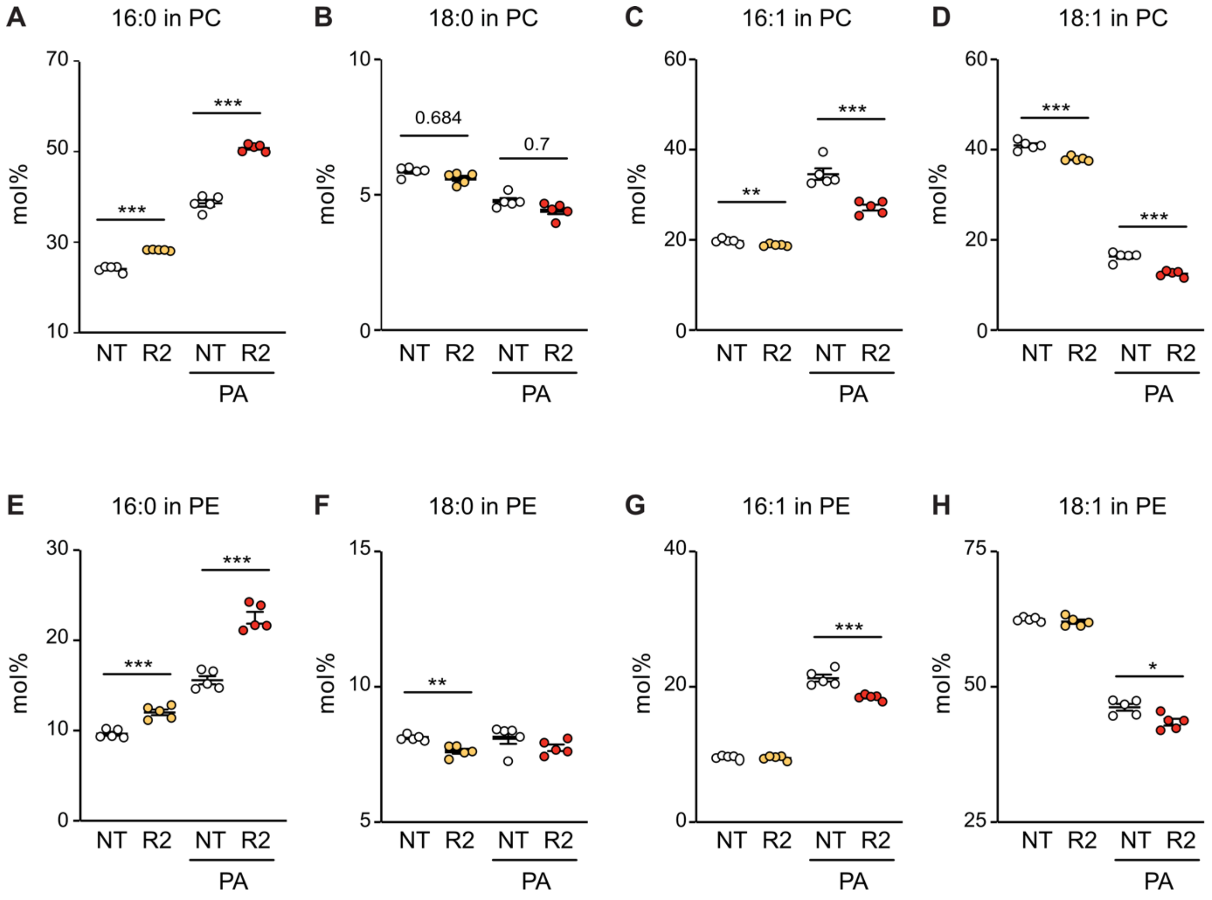

Suppl. Fig. S2. AdipoR2 silencing causes increased levels of SCD substrates (especially 16:0) and depletion of its reaction products (especially 16:1 and 18:1) in phospholipids (data from Exp_190226). (A-H) shows the levels of the indicated fatty acids in phosphatidylcholines (PC) and phosphatidylethanolamines (PE) of HEK293 cells grown in basal media (containing BSA only) or in the presence of 200 µM palmitic acid (PA; conjugated to BSA) and treated with Non-target siRNA (NT) or AdipoR2 siRNA. Significant differences from the NT siRNA control were determined using Student's t-tests with **P*<0.05, ***P*<0.01 and ****P*<0.001.

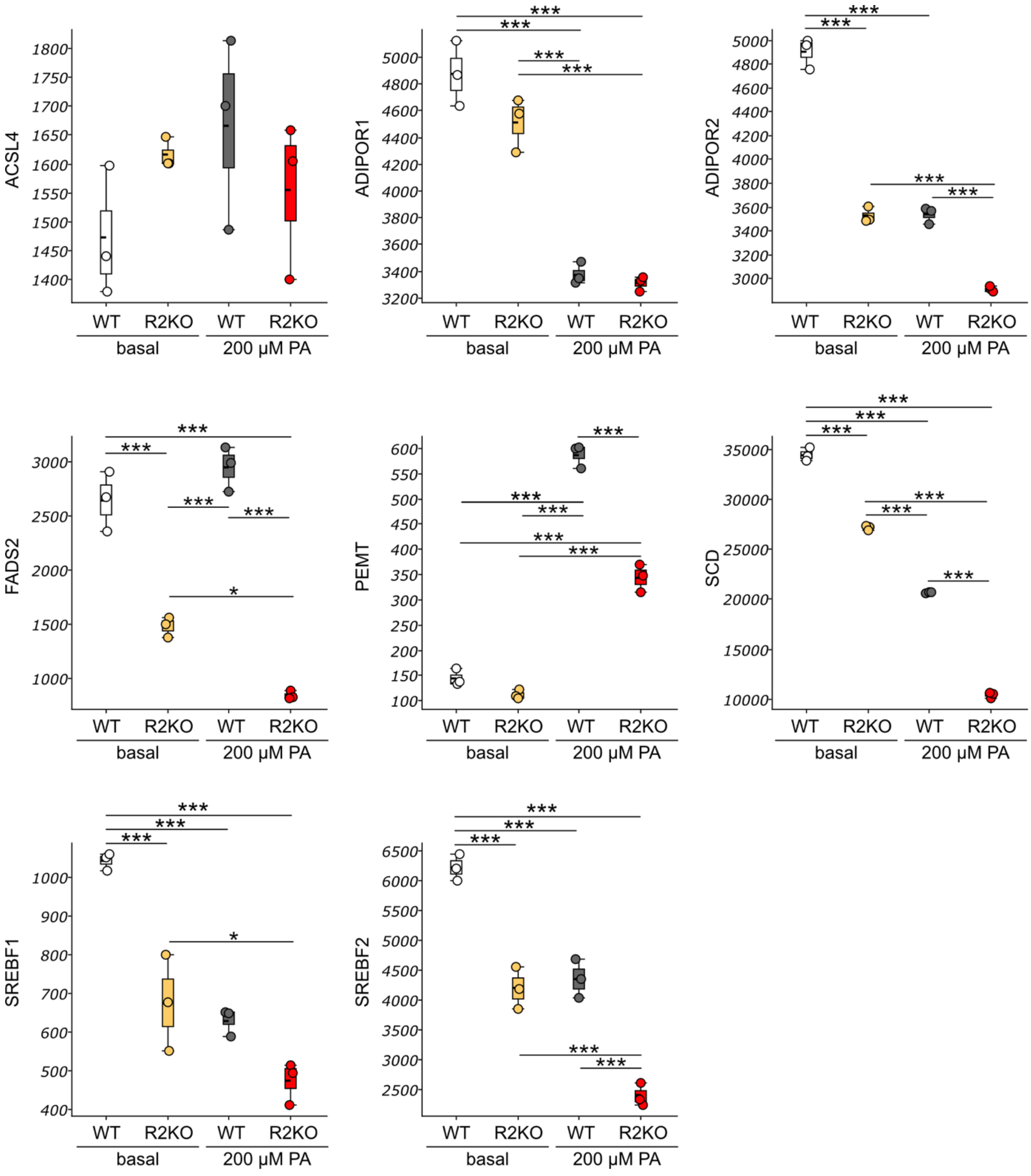

Suppl. Fig. S3. RNAseq data for the genes studied in the present work in HEK293 control and AdipoR2 KO cells. Control (WT) or AdipoR2-KO HEK293 cells (R2KO) were cultivated in serum free basal media or serum free media supplemented with 200 uM PA for 24 hours. RNA was isolated and sequenced. Note that the AdipoR2 transcript is non-functional in the AdipoR2-KO cells. Normalized read counts for each gene are indicated on the y-axis. This is a subset of a previously published RNAseq study [30] that is publicly available at the NCBI GEO repository, accession number GSE158834. *P<0.05, **P<0.01 and ***P<0.001.
